# Comparing the Self-Reported Personality Disorder Traits and Childhood Traumatic Experiences Between Patients With Schizophrenia Vs. Major Depressive Disorder

**DOI:** 10.3389/fpsyt.2021.754174

**Published:** 2021-10-04

**Authors:** Nan Zhao, Dianhong Shi, Juan Huang, Qiuying Chen, Qiang Wang

**Affiliations:** Shanghai Pudong New Area Mental Health Center, Tongji University School of Medicine, Shanghai, China

**Keywords:** affective disorder, psychosis, childhood maltreatment, personality disorder, clinical population

## Abstract

**Introduction:** Personality disorder (PD) and childhood traumatic experience (CTE) are well- recognized risk factors for the development of schizophrenia (SZ) and major depressive disorder (MDD). The relationship between CTE and PD is extremely close, and both conditions can affect subsequent psychiatric disorders. Little is known about the differences of these factors in patients with SZ and those with MDD.

**Materials and Methods:** A total of 1,026 outpatients participated in the study, including 533 (51.9%) with SZ and 493 (48.1%) with MDD who were sequentially sampled. The PD traits were assessed using the Personality Diagnostic Questionnaire Fourth Edition Plus (PDQ-4+). The Child Trauma Questionnaire Short Form (CTQ-SF) was used to assess childhood adversities. The scores and associations of PDQ-4+ and CTQ-SF between patients with SZ and those with MDD were compared.

**Results:** The MDD group exhibited more PD traits and more childhood emotional neglect than the SZ group. In patients with MDD, the correlation between PD traits and CTE was significantly higher than that in patients with SZ. Patients with SZ vs. those with MDD showed different PD traits and CTE. The schizotypal and antisocial PD traits, as well as sexual abuse and physical neglect CTE, were significantly related to SZ. In contrast, the borderline, narcissistic and avoidant PD traits, and emotional abuse/neglect CTE were significantly associated with MDD.

**Discussion:** These findings indicated a robust relationship between CTE and PD traits. Moreover, patients with SZ or MDD, have different interactive patterns. Both CTE and PD traits have the potential to be premorbid risk factors that could be targeted for preventative interventions.

## Introduction

Increasing evidence shows that serious psychiatric disorders, such as schizophrenia (SZ) and major depressive disorder (MDD), are characterized by multiple personality pathology (MPP) (1–3), to a large extent. MPP is an end outcome of childhood traumatic experience (CTE) (4, 5), such as emotional or sexual abuse (6, 7). Personality disorders (PD) often co-occur. The presence of both CTE and PD may result in a longer duration and greater severity of major psychiatric symptoms, such as psychotic or depressive symptoms (8, 9). CTE is a shared risk factor of psychiatric disorders and PD (10). CTE has also been related to the severity and duration of psychiatric disorders in numerous studies (11). These complex relationships are not yet well-elucidated. The current clinical understanding of these correlations is very superficial. Often, only the similarities are seen, which, to a large extent, cover up the differences among psychiatric disorders.

Although SZ is conceptualized as a neurodevelopmental disorder, initial evidence suggests that the presence of PD traits predicts the development of SZ (12, 13). Several lines of evidence have raised the possibility that PD traits may be also associated with the onset of MDD (14, 15). Whether PD traits are associated with the heterogeneity seen in clinical symptoms and categories, and therefore, identify a specific transdiagnostic subgroup, remains unknown (16). Importantly, the relationship between PD traits and CTE is extremely close, and they can both affect subsequent psychiatric disorders. However, it remains unclear whether there is a similar pattern of PD traits and CTE contributing to the development of specific psychiatric disorders.

In this study, we aimed to replicate and build on previous findings on the differences in PD traits and CTE between patients with SZ and those with MDD in a larger sample, and to determine the differences in PD traits and CTE between the two groups. We predict that patients with SZ will have more psychosis-related PD traits, such as paranoid, schizoid, schizotypal PD traits, than patients with MDD. We also hypothesize that patients with MDD will show more affective and anxiety-related PD traits, such as histrionic, narcissistic, borderline, avoidant, dependent, obsessive PD traits, than patients with SZ. We hypothesize that the relationship between PD traits and CTE will vary and may be increased in patients with MDD compared with those with SZ.

## Methods

### Sample and Procedure

The study participants were drawn from a consecutive clinical sample of adult patients, aged 18–60 years, who presented at the Shanghai Pudong New Area Mental Health Center, Tongji University School of Medicine. The hospital's Ethical Committee approved the study, and it was performed according to the Declaration of Helsinki. Informed written consent was provided by patients prior to inclusion in the study. Only patients with SZ or MDD according to the Diagnostic and Statistical Manual of Mental Disorders, 4th edition (DSM-IV), who completed the screening questionnaires, were included in the current analyses. The inclusion criteria were as follows. (1) Age 18–60 years. (2) Completed at least 6 years of education and able to understand the study. (3) Met the DSM-IV criteria for SZ or MDD based on the Structured Clinical Interview for DSM Disorders/Patient edition (SCID-I/P). (4) In a stable treatment condition, that defined as the dose or type of the medication with which the patient has reached psychopathological stability must be constant for at least 2 months prior to inclusion in the study. None of the patients had a history of stroke, brain trauma, central nervous system infection, or seizure. The final sample comprised 1,026 participants (*n* = 533 with SZ and *n* = 493 with MDD). The age and sex distribution between the two groups differed significantly (see Results for further details).

### Self-Reported PD Traits and CTE

The Personality Diagnostic Questionnaire (PDQ-4+) (17), a self-reporting questionnaire, was used to evaluate pathological personality traits, based on DSM-IV criteria. The Chinese version of the PDQ-4+ has been validated as having a high sensitivity (0.89) and moderate specificity (0.65) for screening PD patients (18). The PDQ-4+ screens 12 types of PDs (i.e., paranoid (PAR), schizoid (SCH), schizotypal (SCHT), histrionic (HIS), narcissistic (NAR), borderline (BOR), antisocial (ANT), avoidant (AVO), dependent (DEP), obsessive (OBS), depressive, and negativistic PDs). The depressive and negativistic PDs proposed in the appendix of DSM-IV were not included in the current study. The PDQ-4+ consists of 107 true-false items, including four “too good” questions to prevent participants from undermining the problems and two “suspect questions” to determine whether participants are lying or responding without sincerity. Except for the four “too good” and the two “suspect questions,” “yes” responses to the remaining 93 questions are regarded as pathological responses and counted as 1 point each. Higher subscale scores indicate a greater likelihood of having a certain type of personality disorder.

A quantitative index of childhood maltreatment severity was assessed using the Chinese version of the Child Trauma Questionnaire Short Form (CTQ-SF) (19–21). The CTQ-SF comprises 28 self-report items assessed on five childhood maltreatment subscales: Emotional abuse (EA), physical abuse (PA), sexual abuse (SA), emotional neglect (EN), and physical neglect (PN). The frequency with which each event occurred is rated on a 5-point scale from 1 (never) to 5 (always), with higher scores indicating a higher rate of occurrence. The Chinese version of the CTQ-SF has been confirmed to be a reliable and valid measurement in assessing CTE among Chinese clinical samples (20, 22).

### Statistical Analyses

Statistical analyses were performed on IBM SPSS version 21.0 (IBM Corp., Armonk, NY, USA). One sample Kolmogorov-Smirnov Test was used to examine the data's normal distribution. Demographic characteristics, PD traits, and CTE data for SZ and MD groups were analyzed using frequency and descriptive analyses. Group differences were analyzed using the independent *t*-test or kappa test. Effect sizes were calculated using Cohen's d for mean comparisons. Because some variables do not satisfy the normal distribution, Spearman's correlation analysis was used for PD traits and CTE in the two groups. Lastly, stepwise regression was performed using SZ/MDD as dependent variables, and age, sex, family history, and subscales of PDQ-4+and CTQ-SF as independent variables. Statistical significance was indicated by *p* < 0.05.

## Results

A total of 1,026 outpatients participated in the study, including 533 (51.9%) with SZ and 493 (48.1%) with MDD. Detailed sociodemographic and clinical characteristics between the SZ and MDD groups are reported in Table 1. The two groups did not differ regarding their family history (χ^2^ = 0.347, *p* = 0.556), but significantly differed in age (*t* = 2.730, *p* = 0.006) and sex distribution (χ^2^ = 3.927, *p* = 0.048). The MDD group endorsed more SCH, HIS, NAR, BOR, AVO, DEP, and OBS traits and more childhood emotional neglect than the SZ group (*p* < 0.05).

**Table 1:** Demographic characteristics, personality disorder traits, and childhood traumatic experiences in patients with schizophrenia and major depressive disorder.

**Variables**	**SZ**	**MDD**	**Comparison** ***t/**χ^2^ ^a^**p*****-value effect-size**		
Cases [n (%)]	533 (51.9)	493 (48.1)	–	–	–
Age (years) [Mean (SD)]	29.7 (9.1)	31.3 (9.9)	2.730	**0.006**	–
Male [*n* (%)]	228 (42.8)	181 (36.7)	3.927	**0.048**	–
Family history [*n* (%)]	59 (11.1)	49 (9.9)	0.347	0.556	–
Personality disorder traits by PDQ-4+ [Mean (SD)]					
Paranoid personality disorder	3.3 (1.7)	3.4 (1.7)	1.139	0.255	−0.059
Schizoid personality disorder	2.7 (1.6)	2.9 (1.6)	2.215	**0.027**	−0.125
Schizotypal personality disorder	4.5 (1.9)	4.3 (1.0)	1.504	0.133	0.138
Histrionic personality disorder	3.8 (1.9)	4.1 (1.7)	2.700	**0.007**	−0.167
Narcissistic personality disorder	3.7 (2.0)	4.1 (2.0)	3.938	**<0.001**	−0.2
Borderline personality disorder	4.8 (2.3)	5.5 (2.2)	5.195	**<0.001**	−0.311
Antisocial personality disorder	2.2 (1.7)	2.0 (1.8)	1.638	0.102	0.114
Avoidant personality disorder	4.2 (1.7)	4.7 (1.7)	4.657	**<0.001**	−0.294
Dependent personality disorder	3.9 (1.9)	4.1 (2.0)	2.168	**0.030**	−0.103
Obsessive-compulsive personality disorder	4.2 (1.8)	4.5 (1.7)	2.946	**0.003**	−0.171
Childhood traumatic experiences by CTQ-SF [Mean (SD)]					
Emotional abuse	7.7 (3.1)	7.9 (3.4)	0.843	0.400	−0.062
Physical abuse	6.3 (2.3)	6.2 (2.3)	1.012	0.312	0.043
Sexual abuse	6.1 (2.1)	5.8 (1.8)	2.725	**0.007**	0.154
Emotional neglect	12.3 (4.7)	12.9 (5.1)	1.970	**0.049**	−0.122
Physical neglect	9.3 (3.0)	8.9 (3.2)	1.836	0.067	0.129
Total scores of abuse and neglect	41.6 (10.1)	41.5 (10.2)	0.089	0.929	0.010

*^a^t/χ^2^: t for the independent t-test; χ^2^ for kappa test. SZ, schizophrenia; MDD, major depressive disorder; PDQ-4+, Personality Diagnostic Questionnaire Fourth Edition Plus; CTQ-SF, Child Trauma Questionnaire Short Form. Bold in significant*.

Associations between all variables of interest (PDQ-4+ and CTQ-SF) were tested. Non-parametric Spearman correlation analysis yielded significant correlations between different types of PD traits (Table 2) and different types of CTE (Table 3).

**Table 2:** Correlations of different types of personality disorder traits in patients with schizophrenia and those with major depressive disorder.

**SZ (r)**	**PAR**	**SCH**	**SCHT**	**HIS**	**NAR**	**BOR**	**ANT**	**AVO**	**DEP**	**OBS**
PAR	1.000	0.112^**^	0.531^**^	0.302^**^	0.625^**^	0.514^**^	0.331^**^	0.321^**^	0.297^**^	0.430^**^
SCH	–	1.000	0.188^**^	0.065	0.104^*^	0.109^*^	0.069	0.120^**^	0.128^**^	0.112^**^
SCHT	–	–	1.000	0.402^**^	0.499^**^	0.575^**^	0.392^**^	0.321^**^	0.338^**^	0.445^**^
HIS	–	–	–	1.000	0.529^**^	0.602^**^	0.543^**^	0.182^**^	0.398^**^	0.354^**^
NAR	–	–	–	–	1.000	0.565^**^	0.382^**^	0.413^**^	0.398^**^	0.579^**^
BOR	–	–	–	–	–	1.000	0.594^**^	0.388^**^	0.480^**^	0.409^**^
ANT	–	–	–	–	–	–	1.000	0.223^**^	0.340^**^	0.272^**^
AVO	–	–	–	–	–	–	–	1.000	0.466^**^	0.406^**^
DEP	–	–	–	–	–	–	–	–	1.000	0.387^**^
OBS	–	–	–	–	–	–	–	–	–	1.000
**MDD (r)**	**PAR**	**SCH**	**SCHT**	**HIS**	**NAR**	**BOR**	**ANT**	**AVO**	**DEP**	**OBS**
PAR	1.000	0.217^**^	0.514^**^	0.209^**^	0.487^**^	0.493^**^	0.325^**^	0.364^**^	0.252^**^	0.329^**^
SCH	–	1.000	0.328^**^	−0.047	0.072	0.263^**^	0.103^*^	0.376^**^	0.171^**^	0.163^**^
SCHT	–	–	1.000	0.200^**^	0.396^**^	0.498^**^	0.383^**^	0.392^**^	0.333^**^	0.364^**^
HIS	–	–	–	1.000	0.419^**^	0.415^**^	0.404^**^	0.043	0.268^**^	0.202^**^
NAR	–	–	–	–	1.000	0.435^**^	0.366^**^	0.253^**^	0.199^**^	0.379^**^
BOR	–	–	–	–	–	1.000	0.534^**^	0.400^**^	0.426^**^	0.293^**^
ANT	–	–	–	–	–	–	1.000	0.114^*^	0.191^**^	0.097^*^
AVO	–	–	–	–	–	–	–	1.000	0.510^**^	0.303^**^
DEP	–	–	–	–	–	–	–	–	1.000	0.245^**^
OBS	–	–	–	–	–	–	–	–	–	1.000

*SZ, schizophrenia; MDD, major depressive disorder; PAR, Paranoid; SCH, Schizoid; SCHT, Schizotypal; HIS, Histrionic; NAR, Narcissistic; BOR, Borderline; ANT, Antisocial; AVO, Avoidant; DEP, Dependent; OBS, Obsessive-compulsive*.

* *Correlation is significant at the 0.05 level (2-tailed)*.

** *Correlation is significant at the 0.01 level (2-tailed). Bold in significant*.

**Table 3:** Correlations of different types of childhood traumatic experiences in patients with schizophrenia and those with major depressive disorder.

**SZ (r)**	**EA**	**PA**	**SA**	**EN**	**PN**
EA	1.000	0.392^**^	0.400^**^	0.245^**^	0.194^**^
PA	–	1.000	0.321^**^	0.185^**^	0.176^**^
SA	–	–	1.000	0.130^**^	0.138^**^
EN	–	–	–	1.000	0.383^**^
PN	–	–	–	–	1.000
**MDD (r)**	**EA**	**PA**	**SA**	**EN**	**PN**
EA	1.000	0.543^**^	0.338^**^	0.396^**^	0.336^**^
PA	–	1.000	0.272^**^	0.237^**^	0.201^**^
SA	–	–	1.000	0.119^**^	0.115^*^
EN	–	–	–	1.000	0.454^**^
PN	–	–	–	–	1.000

*SZ, schizophrenia; MDD, major depressive disorder; EA, Emotional abuse; PA, Physical abuse; SA, Sexual abuse; EN, Emotional neglect; PN, Physical neglect*.

* *Correlation is significant at the 0.05 level (2-tailed)*.

** *Correlation is significant at the 0.01 level (2-tailed). Bold in significant*.

In patients with SZ, the PAR and SCHT PD traits were significantly associated with the EA type of CTE. In patients with MDD, the correlation between PD and CTE was more significant than that in patients with SZ (Table 4).

**Table 4:** Correlations of different types of personality disorder traits in patients with schizophrenia and those with major depressive disorder.

**SZ r(p)**	**PAR**	**SCH**	**SCHT**	**HIS**	**NAR**	**BOR**	**ANT**	**AVO**	**DEP**	**OBS**
EA	0.112^**^	0.011	0.132^**^	0.024	0.101^*^	0.062	0.108^*^	0.000	0.057	0.042
PA	0.018	−0.037	0.022	0.036	0.044	0.034	0.117^**^	−0.057	−0.011	−0.036
SA	0.070	0.002	0.104^*^	0.056	0.083	0.081	0.117^**^	0.011	0.081	0.026
EN	0.085	0.036	0.071	0.043	0.062	0.049	0.028	−0.015	−0.067	0.005
PN	0.036	0.026	−0.003	−0.002	0.057	0.016	0.001	−0.025	0.020	0.004
**MDD r(p)**	**PAR**	**SCH**	**SCHT**	**HIS**	**NAR**	**BOR**	**ANT**	**AVO**	**DEP**	**OBS**
EA	0.198^**^	0.074	0.144^**^	0.124^**^	0.195^**^	0.206^**^	0.266^**^	0.043	0.078	0.023
PA	0.159^**^	0.062	0.124^**^	0.048	0.127^**^	0.137^**^	0.193^**^	−0.015	0.020	0.024
SA	0.110^*^	−0.025	0.059	0.161^**^	0.103^*^	0.194^**^	0.198^**^	−0.041	0.053	0.010
EN	0.090^*^	0.080	0.098^*^	0.052	0.038	0.083	0.083	0.064	0.023	−0.009
PN	0.054	0.106^*^	0.118^**^	0.038	−0.030	0.062	0.074	0.025	0.054	0.014

*SZ, schizophrenia; MDD, major depressive disorder; PAR, Paranoid; SCH, Schizoid; SCHT, Schizotypal; HIS, Histrionic; NAR, Narcissistic; BOR, Borderline; ANT, Antisocial; AVO, Avoidant; DEP, Dependent; OBS, Obsessive-compulsive; EA, Emotional abuse; PA, Physical abuse; SA, Sexual abuse; EN, Emotional neglect; PN, Physical neglect*.

* *Correlation is significant at the 0.05 level (2-tailed)*.

** *Correlation is significant at the 0.01 level (2-tailed). Bold in significant*.

As shown in Table 5, forward stepwise logistic regression was used to identify which of the PDQ-4+ and CTQ-SF variables were most strongly related to the SZ and MDD diagnosis. The SZ/MDD was listed as the dependent variable, while age, sex, family history, and the PDQ-4+ and CTQ-SF variables were listed as independent variables. The PD variables of SCHT (*p* < 0.001) and ANT (*p* < 0.001) were significantly related to SZ. In contrast, the BOR (*p* < 0.001), NAR (*p* = 0.006), AVO (*p* = 0.009) PD traits were significantly associated with MDD in this model. The CTE variables of SA (*p* = 0.004) and PN (*p* = 0.005) were significantly related to SZ, while EA (*p* = 0.017) and EN (*p*= 0.028) were significantly related MDD.

**Table 5:** Forward stepwise regression for distinguishing between patients with schizophrenia and major depressive disorder.

**Variables in Model**	**Beta**	**S.E**.	**Odds ratio**	**95% CI**	**Wald statistic**	***p*-value**
Age	0.020	0.007	1.020	1.006–1.035	7.436	0.006
SCHT	−0.239	0.044	0.788	0.723–0.858	29.665	0.000
ANT	−0.231	0.049	0.793	0.720–0.874	21.968	0.000
BOR	0.277	0.043	1.320	1.212–1.437	40.772	0.000
NAR	0.113	0.041	1.119	1.033–1.213	7.629	0.006
AVO	0.116	0.044	1.123	1.029–1.225	6.772	0.009
EA	0.061	0.025	1.062	1.011–1.117	5.730	0.017
SA	−0.112	0.039	0.894	0.828–0.966	8.091	0.004
EN	0.035	0.016	1.036	1.004–1.068	4.799	0.028
PN	−0.070	0.025	0.932	0.887–0.979	7.854	0.005

*SCHT, Schizotypal; ANT, Antisocial; BOR, Borderline; NAR, Narcissistic; AVO, Avoidant; EA, Emotional abuse; SA, Sexual abuse; EN, Emotional neglect; PN, Physical neglect*.

## Discussion

### Major Findings

The primary aim of the present study was to examine the difference and relationship between PD traits and CTE within two major psychiatric diagnostic groups, namely, SZ and MDD. We noted three key findings. First, patients with MDD reported more PD traits (7 in 10 PD domains) than those with SZ. Second, the correlation between PD traits and CTE was more obvious in the MDD group than in the SZ group. Third, SZ and MDD are associated with different PD traits and CTE. These findings indicated that a robust relationship exists between CTE and PD traits in patients with SZ and those with MDD, with different interactive patterns. To the best of our knowledge, this is the first study conducted in a large Chinese clinical sample to establish and compare the associations between CTE and PDs between SZ and MDD.

### PD Traits

In this study, patients with MDD reported increased PD traits compared to patients with SZ, even in Cluster A PD traits (i.e., schizoid PD) that are marked by odd and eccentric behaviors. These results are in line with those of Zhang et al. (18), who found that nearly 42.18% of the cases with mood disorders were associated with at least one DSM-IV PD and that the comorbidity rate was higher than that found in patients with SZ (24.00%). The high comorbidity rate of 42.36% was also reported by Zheng et al. for 258 of 609 patients with MDD who met at least one criterion of diagnosis for the DSM-IV PD (2). Ryo et al. (23) explored the role of personality traits, childhood abuse, and depressive symptoms in patients with SZ. They found that personality traits mediate the relationship between childhood abuse and depressive symptoms, and that such mediator effect could occur independently of the type of psychiatric disorders. In other words, PD traits may play a more important role in the relationship between CTE and MDD (24), than between CTE and SZ. From the perspective of clinical intervention, it may be of more significance to systematically understand CTE and PD traits for psychological intervention of patients with MDD than SZ.

### CTE

EN reported by the participants were more frequent in the patients with MDD than those with SZ, as confirmed through a recent meta-analysis (11). Surprisingly, patients with SZ endorsed SA more than patients with MDD. Consistent with an umbrella review by Helen et al. (25), they found that there is high-quality evidence supporting the association between childhood SA and two psychiatric disorders: SZ and post-traumatic stress disorder. Other studies have found that the association of SA with schizophrenia is not consistent (26–28). The current retrospective study cannot conclude that SA is a causal factor of SZ, but it does not rule out the possibility. At the very least, the evidence suggests that victims of childhood adversities are more vulnerable to particular kinds of stress in many ways and that the adverse effects can be long-lasting (29). We also note that emotional abuse and neglect elevate susceptibility to MDD. Emotional abuse and neglect, defined as recurrent parental critical attacks, rejection, devaluation, contempt, and ignoring the child. An implication of this perspective is that emotional maltreatment should have significant consequences for depression development, impacting not only on the personality organization but also on the individual's mood states.

### Associations Between PD Traits and CTE

Interestingly, significant associations were mainly between childhood abuse and cluster A PD traits in the SZ group. Moreover, all types of CTE and Cluster B (including BOR, HIS, NAR, ANT, are characterized by dramatic and unpredictable behaviors) and Cluster A PD traits were significantly related in the MDD group. Previous evidence (30, 31) has suggested possible mediating effects of PD traits between childhood adversities and psychiatric disorders. Our results further determined that this intermediate effect may be more focused in related PD traits corresponding to specific psychiatric disorders (i.e., Cluster A PDs to SZ, Cluster B PDs to MDD). Such correlation patterns were confirmed by our regression analysis. This suggests that the effects of CTE and PD traits are different in different psychiatric disorders. For example, MDD and SZ may result from two pathways with different types of CTE and PD traits contributing: (1) SZ: childhood SA and PN to Cluster A PD traits to SZ, and (2) MDD: childhood EA and EN to Cluster B PD traits to MDD. However, it is very difficult to be certain if CTE acts as a general stressor for PD and psychiatric disorders, or if CTE is a causative factor with a more genetic influence in its development?

### Limitations

Several limitations should be considered when interpreting our findings. The patients with SZ were significantly younger than those with MDD (by an average of ~1–2 years). Thus, there may be potential selection bias and memory information bias, as younger patients would be more likely to recall and report CTE than the elderly. Second, PD traits and CTE were assessed using a self-report, retrospective measure. Self-report measures assume reliable reporting, which may be a concern in our patient populations, especially in patients with SZ, who are more likely to have cognitive impairment (32–34). Furthermore, the data are cross-sectional, cohort studies are more valuable for establishing a temporal link between CTE, PD traits, and later psychiatric disorders. Finally, this is a single-center study, and our sample may not represent the entire Chinese population. Consequently, the generalizability of our findings may be limited. However, the single-site design may have increased sample homogeneity and diagnostic consistency.

## Conclusions

Our results show that most PD traits and childhood emotional adversities were more prominent in patients with MDD. In contrast, childhood sexual and physical adversities are more prominent in patients with SZ. The history of CTE is significantly related to specific PD traits of the disease spectrum, such as SCH and SCHT PD traits in the psychotic spectrum, and BOR and NAR PD traits in the affective spectrum. Therefore, both CTE and PD traits have the potential to be premorbid risk factors that could be targeted for preventative interventions, including cognitive behavioral therapy (35).

## Data Availability Statement

The original contributions presented in the study are included in the article/supplementary material, further inquiries can be directed to the corresponding author/s.

## Ethics Statement

The studies involving human participants were reviewed and approved by Ethical Committee of Shanghai Pudong New Area Mental Health Center. The patients/participants provided their written informed consent to participate in this study. Written informed consent was obtained from the individual(s) for the publication of any potentially identifiable images or data included in this article.

## Author Contributions

NZ collected the data and performed the statistical analyses. DS wrote the original manuscript. JH and QC designed and wrote the study protocol. QW offered many important suggestions on this study. All authors contributed to the article and approved the submitted version.

## Funding

This study was supported from Excellent Young Medical Talents Training of Health Commission of Pudong New Area (PWRq2020-49); Pudong New Area of Science and Technology Development Fund (PKJ2019-Y25); Shanghai Municipal Health Industry Clinical Research Project (2020Y194).

## Conflict of Interest

The authors declare that the research was conducted in the absence of any commercial or financial relationships that could be construed as a potential conflict of interest.

## Publisher's Note

All claims expressed in this article are solely those of the authors and do not necessarily represent those of their affiliated organizations, or those of the publisher, the editors and the reviewers. Any product that may be evaluated in this article, or claim that may be made by its manufacturer, is not guaranteed or endorsed by the publisher.
